# Brain-First vs. Body-First Models in Neurodegenerative Disease: A Perspective Review

**DOI:** 10.3390/neurosci7030057

**Published:** 2026-05-08

**Authors:** Giuseppe Forte, Maria Casagrande

**Affiliations:** Department of Dynamic, Clinical Psychology and Health Studies, “Sapienza” University of Rome, 00185 Rome, Italy; maria.casagrande@uniroma1.it

**Keywords:** brain-first, body-first, neurodegenerative diseases, dementia, biomarkers, autonomic dysfunction, disease trajectories, phenotypic heterogeneity, narrative review

## Abstract

Recent advances in neurodegenerative disease research increasingly support the existence of multiple trajectory signatures underlying the heterogeneity of cognitive decline syndromes. Originally proposed in Parkinson’s disease, the brain-first and body-first models have emerged as conceptual frameworks to explain variability in disease onset, prodromal features, and progression across dementia-related disorders. Brain-first phenotypes are defined by the early emergence of central nervous system pathology and cognitive symptoms, whereas body-first phenotypes are characterized by prominent peripheral or autonomic dysfunctions that precede central involvement. Within this perspective, neurodegeneration is not viewed as a uniform, brain-restricted process, but a dynamic interaction between central, peripheral, and network-level mechanisms. Integrating central and peripheral biomarkers, autonomic physiology, and alterations in functional connectivity provides a coherent framework for interpreting phenotypic heterogeneity and prodromal dysregulation across dementia syndromes. Current evidence supporting brain-first and body-first trajectories is largely associative, and their clinical translation requires rigorous validation. Accordingly, this narrative perspective review aims to provide a critical and integrative conceptual framework that synthesizes existing evidence, identifies unresolved questions, and outlines research priorities for trajectory-based stratification, rather than offering a definitive diagnostic classification or pharmacological evaluation. The present work adopts a conceptual and mechanistic perspective rather than a clinically prescriptive or trial-oriented one. Its aim is to articulate a generative framework capable of producing testable hypotheses about disease trajectories and biomarker constellations across neurodegenerative syndromes.

## 1. Introduction

Neurodegenerative diseases are a heterogeneous group of pathologies that are unified by progressive neurodegeneration, which commonly results in widespread cognitive deterioration and heterogeneous non-cognitive neurological phenomena [1,2] even though standard models have primarily attributed central nervous system (CNS) pathology to be the sole cause of neurodegeneration, the newly emerging body of evidence emphasizes the key role played by the peripheral nervous system (PNS)—more specifically by the autonomic nervous system (ANS)—in disease development and disease progression [3,4,5,6,7,8,9,10,11,12].

A progressively sophisticated understanding has established a conceptually central model that separates brain-first and body-first trajectory signatures. This model provides a comprehensive account of disease heterogeneity and progression, in contrast to traditional unitary models, and may potentially transform our approach to classification and intervention [13,14,15,16,17,18]. Operationally, brain-first phenotypes are defined by the temporal precedence of central pathology and cognitive prodromes, while body-first phenotypes are defined by the early appearance of peripheral/autonomic dysfunction preceding central involvement (See Table 1).

According to the brain-first hypothesis, the neurodegenerative pathologies are hypothesized to originate primarily in intrinsic regions of the central nervous system (CNS), such as the limbic system, basal ganglia, and neocortex [19]. Local onset inevitably leads to the initial onset of cognitive, motor, and behavioral symptoms, indicating regional CNS disease [20]. In this case, misfolded proteins (e.g., alpha-synuclein, tau, or amyloid-β) accumulate and propagate by highly specific, interconnected networks of neurons, in keeping with both classic and contemporary network degeneration theories [20,21,22,23]. Consequently, this form of central onset is associated with the relatively rapid development of cardinal clinical signs, with peripheral involvement being either significantly absent or subtle in the early stages of the disease.

In contrast, the body-first model emphasizes the peripheral nervous system (PNS), namely the autonomic nervous system (ANS) and the enteric nervous system (ENS), as the primary location of disease onset. In this model, abnormal proteins are hypothesized to emerge in tissues outside of the cerebrum (e.g., gut, olfactory epithelium, or cardiac plexus) and then spread through retrograde axonal transport on autonomic efferent and afferents, e.g., vagus nerve and sympathetic chains, ultimately invading the brainstem and then migrating to more rostral cortical regions [24,25,26]. These pathogenic spread manifests clinically as prominent prodromal non-motor symptoms, including constipation, orthostatic hypotension, lower heart rate variability, often predating hallmark central symptoms by several years [6,27,28,29]. The early and significant involvement of the ANS in this model compels a re-evaluation of neurodegeneration through a holistic, multisystemic lens, thereby challenging the long-standing CNS-centric view.

Support for brain-first and body-first trajectories arises from several complementary sources of evidence. Neuropathological studies, particularly staging work, demonstrate where pathological proteins appear first. For instance, α-synuclein may accumulate initially in limbic or cortical regions (consistent with a brain-first hypothesis) or, alternatively, in the enteric nervous system and autonomic ganglia (consistent with a body-first hypothesis). Neuroimaging studies provide converging in vivo evidence by identifying the earliest sites of structural or functional change, such as hippocampal atrophy and cortical hypometabolism in brain-first cases versus early brainstem or nigrostriatal involvement in body-first variants. Peripheral tissue analyses, including skin and gut biopsies, can detect misfolded proteins years before overt symptoms and are particularly informative for identifying body-first trajectories. Autonomic function testing, such as measurements of heart rate variability, orthostatic blood pressure responses, or sudomotor activity, suggests early autonomic nervous system dysfunctions, again pointing to peripheral initiation when present before cognitive symptoms.

This dualistic model significantly redefines our understanding, suggesting disease-specific boundaries (see above) and being a “rediscovery” of the body’s inseparable nature in neurodegenerative disease. Peripheral symptoms are no longer merely epiphenomena but are comprehended, via the brain-first and body-first models, as signs of ANS and PNS activity as essential indicators of disease initiation and driving forces [13]. Nevertheless, important limitations must be acknowledged at the outset: heterogeneity within individual diseases complicates clear classification, selection biases in research samples may skew the findings, much of the literature is based on cross-sectional data rather than longitudinal follow-up, and treatment or comorbidity-related confounding is a frequent issue.

This profound paradigm shift has distant implications for presymptomatic diagnosis since new peripheral biomarkers and measures of autonomic dysfunction potentially give critical windows of therapeutic opportunity before irreversible CNS damage occurs. In addition, it illuminates promising pathways for pathology-guided treatments that target peripheral mechanisms with the capability to reverse the natural course of these disabling disorders. Combined, the brain-first and body-first strategy provides an integrative model that harmonizes central and peripheral pathogenic inputs. This broad generalization not only underscores the great heterogeneity of neurodegenerative disorders but also calls for a shift to a wide conceptualization of disease mechanisms.

Against this background, the present narrative perspective review (see Appendix A for methodological approach) pursues three primary aims. First, it critically compares brain-first and body-first neurodegenerative trajectories across major neurodegenerative disorders, focusing on differences in sites of initiation, propagation pathways, and prodromal clinical features. Second, it integrates evidence from central and peripheral biomarkers, autonomic physiology, and network-level dysfunctions into a unified conceptual framework capable of explaining phenotypic heterogeneity beyond traditional disease-based classifications. Third, it outlines key research priorities aimed at early stratification, longitudinal validation of disease trajectories, and phenotype-guided study design.

At the same time, the brain-first and body-first distinction remains a provisional and evolving framework. Much of the supporting evidence is cross-sectional, surrogate-based, or indirectly inferred, and competing interpretations remain plausible. Accordingly, this review adopts a perspective that emphasizes uncertainty, model limitations, and the need for validation, suggesting a conceptual integration and illustrative examples that clarify trajectory-based models of neurodegeneration. Beyond a descriptive distinction, the brain-first/body-first framework is used here as a comparative mechanistic model. Rather than cataloguing syndromes, the aim of this review is to focus on how differences in sites of initiation are expected to shape propagation dynamics, biomarker constellations, network vulnerability, and windows of phenotypic instability. Therefore, the goal is to articulate trajectory-specific signatures that can be prospectively tested, rather than proposing diagnostic criteria or therapeutic prescriptions. To achieve true comparative integration, the present framework is structured along four analytical dimensions: (1) site of initiation (central vs. peripheral), (2) propagation dynamics (network-constrained vs. autonomic ascending pathways), (3) temporal sequencing of biomarker constellations, and (4) system-level interactions including autonomic and body–brain coupling mechanisms. These dimensions allow brain-first and body-first trajectories to be compared as dynamic configurations rather than static categories, enabling a more rigorous cross-disease interpretation of heterogeneity.

## 2. Specific Dementia Syndromes in the Context of Brain-First and Body-First Models

In presenting these syndromes, the goal is not merely descriptive. The comparative analysis of these disorders serves to illustrate how the brain-first/body-first framework provides a coherent interpretative lens through which to understand their divergent biological pathways and clinical presentations. This cross-syndromic perspective suggests that heterogeneity in dementia is not random, but reflects distinct initiating sites and trajectory signatures that can be systematically mapped and leveraged for diagnostic refinement.

### 2.1. Alzheimer’s Disease: Predominantly Brain-First, with Emerging Body-First Modifiers

Alzheimer’s disease (AD) has long been conceptualized as the quintessential brain-first neurodegenerative disorder, primarily hypothesized to originate within central neural circuits. Classical neuropathological staging [1,2,14,30,31] places the genesis of disease in the medial temporal lobe (particularly the hippocampal formation), where extracellular deposits of amyloid-β (Aβ) plaques and intraneuronal tau neurofibrillary tangles first emerge. These early changes propagate along highly interconnected neural networks, spreading into widespread neocortical territories. This early involvement of medial temporal lobe structures correlates with the memory impairment characteristic of mild cognitive impairment (MCI) progressing to AD dementia and maps closely into the clinical course: patients typically present with an amnestic form of mild cognitive impairment (aMCI), followed by progressive involvement of additional cognitive domains and eventual transition to fully established dementia [32,33,34]. The centrality of this brain-first pathogenic cascade is reinforced by converging biomarker evidence [35]. Structural and functional neuroimaging further demonstrate temporoparietal hypometabolism and cortical atrophy, particularly in hippocampal and associative regions, as well as early hippocampal atrophy in MCI patients destined for AD [36]. Functional PET scans may detect amyloid and tau deposition in limbic and neocortical regions before clinical decline [37]. Furthermore, neuroinflammation and synaptic loss in these areas drive cognitive deterioration [38]. Such biomarkers form the backbone of the AT(N) framework, highlighting amyloid (A), tau (T), and neurodegeneration (N) as core indicators of central disease biology.

Recent plasma p-tau assays (e.g., p-tau181, p-tau217, p-tau231) provide peripheral readouts of central tau pathology.

At the molecular level, the prevailing amyloid cascade hypothesis posits that an imbalance between Aβ production and clearance initiates the pathogenic sequence, triggering downstream tau hyperphosphorylation, synaptic dysfunction, and neuronal death. Genetic evidence lends strong support to this brain-centered model: mutations in APP, PSEN1, and PSEN2 invariably lead to early-onset familial AD, while the APOE ε4 allele constitutes the strongest genetic risk factor for late-onset AD, promoting Aβ aggregation and impairing clearance.

Nevertheless, peripheral and systemic factors are increasingly recognized as modulators of this central pathology. Evidence has associated gut microbiota dysbiosis, systemic inflammation, and altered immune responses to amyloid and tau pathology through the gut–brain axis [39]. Peripheral metabolic disturbances, such as insulin resistance and vascular dysfunction, are also implicated as accelerators of central pathology. However, these associations remain preliminary: effect sizes are often small, confounding variables are significant, and reverse causation cannot be excluded. Accordingly, while AD remains predominantly a brain-first disorder, peripheral influences are more appropriately conceptualized as emerging modifiers of central pathology.

### 2.2. Dementia with Lewy Bodies: Body-First and Brain-First Phenotypes Within a Spectrum

Dementia with Lewy bodies represents the most compelling example of a disorder that spans the continuum between body-first and brain-first origins. Traditionally, the disease has been understood as a body-first syndrome in which misfolded α-synuclein accumulates initially in the autonomic and enteric nervous systems, including the gut, salivary glands, and olfactory mucosa, before significant cortical involvement, and subsequently propagates centrally via the vagus nerve and sympathetic chains, supporting a peripheral-to-central trajectory of pathology [14,16,40]. This mechanism is clinically reflected in prodromal symptoms such as hyposmia and autonomic dysfunction, which often precede cognitive decline by years [14,16,40]. Nevertheless, recent clinical, neuropathological, and imaging evidence has highlighted the existence of brain-first phenotypes, characterized by early deposition of α-synuclein within limbic and cortical regions [13]. In these cases, attentional deficits, visuospatial dysfunctions, neuropsychiatric symptoms, and hallucinations emerge as the first signs of disease, while peripheral involvement appears later or remains comparatively mild [41]. The duality between body-first and brain-first trajectories carries profound clinical consequences. Body-first patients often display a long prodromal course dominated by non-cognitive disturbances, followed by an abrupt transition to dementia once cortical spread is established, often with severe fluctuations and marked visuospatial impairment [42]. By contrast, brain-first patients may initially resemble atypical presentations of Alzheimer’s disease, with posterior cortical deficits and attentional disturbances preceding autonomic failure [17]. These divergent clinical paths are mirrored by distinct biomarker and imaging profiles, with body-first showing early alterations in brainstem and autonomic structures, while brain-first cases exhibit posterior cortical hypometabolism and sometimes Alzheimer-like signatures [13].

### 2.3. Parkinson’s Disease Dementia: Dual Body-First and Brain-First Pathogenesis

Parkinson’s disease and its associated dementia further illustrate the coexistence of body-first and brain-first pathogenic trajectories [13,14,15,16,17,18]. The body-first phenotype typically presents with early autonomic dysfunction, while the brain-first subtype is more strongly associated with asymmetric motor onset and later autonomic features [13,14,15,16,17,18]. This hypothesis is supported by the frequent presence of non-motor symptoms such as constipation and hyposmia, which may precede the motor syndrome by decades. The subsequent spread of α-synuclein to limbic and cortical regions underlies the transition to dementia, which often develops more rapidly in this subgroup. By contrast, brain-first Parkinson’s disease is characterized by initial pathology in central structures such as the amygdala or substantia nigra, leading to an earlier predominance of motor features, while non-motor and cognitive symptoms appear later and progress more slowly. This brain-first trajectory has been linked to the selective vulnerability of dopaminergic neurons in the substantia nigra pars compacta, with misfolded α-synuclein aggregates forming Lewy bodies and Lewy neurites that propagate along interconnected basal ganglia and cortical circuits [43]. These two routes have profound implications for the heterogeneity of clinical outcomes. Patients with body-first Parkinson’s disease generally experience a longer prodromal phase, but once cortical involvement occurs, they face a higher likelihood of developing early cognitive decline and dementia. In contrast, brain-first patients often follow a more classical parkinsonian trajectory with slower cognitive deterioration. Neuroimaging studies have confirmed these differences, showing widespread autonomic and brainstem involvement in body-first cases, while brain-first patients exhibit more localized nigrostriatal degeneration in the initial stages [13,14,15,16,17,18]. Such distinctions underscore the need for tailored biomarkers and personalized interventions: colonic or skin biopsies and cardiac sympathetic imaging may be especially useful in identifying body-first disease, whereas nigrostriatal dopaminergic imaging and central fluid biomarkers are more informative in brain-first cases. The recognition of these dual trajectories situates Parkinson’s disease dementia within a broader spectrum of synucleinopathies, where peripheral and central origins coexist and interact to shape the risk and timing of cognitive decline.

### 2.4. Frontotemporal Dementia: Prototypical Brain-First Neurodegeneration

Frontotemporal dementia represents a disorder where pathology is largely confined to frontal and temporal cortical regions. Frontotemporal dementia represents a prototypical brain-first neurodegenerative disease where pathogenic protein inclusions—such as tau, TDP-43, or FUS—initially accumulate in the frontal and anterior temporal lobes, producing early deficits in executive, social, and language functions [44,45,46]. Clinically, this results in characteristic syndromes, such as the behavioral variant, characterized by profound changes in personality, judgment, and social conduct, and the language variants, marked by progressive aphasia. Neuroimaging consistently demonstrates focal atrophy in these brain-first regions, while genetic studies support the view that intrinsic central nervous system mechanisms drive the disease [44,45,46]. Unlike Alzheimer’s disease, Parkinson’s disease, or dementia with Lewy bodies, there is currently no robust evidence for peripheral initiation; however, systemic modulators (immune/metabolic) may influence expression. For example, autonomic involvement has been reported in some behavioral-variant FTD cases, suggesting that peripheral systems may modulate disease expression, even if not the primary initiator [44,45,46]. This renders FTD a paradigmatic example of a disorder rooted in the central nervous system, whose clinical and biological characteristics align seamlessly with a brain-first model.

### 2.5. Vascular Contributions and Mixed Dementia Phenotypes

The interplay between vascular pathology and neurodegeneration adds further complexity to the brain-first and body-first framework. For vascular dementia (VaD), the body-first perspective shifts toward systemic risk factors. Peripheral conditions, such as hypertension, diabetes, dyslipidemia, and chronic systemic inflammation, drive cerebrovascular pathology, white matter damage, and small vessel disease. Although not a proteinopathy in the strict sense, VaD exemplifies how peripheral vascular and metabolic insults precede and determine central neurodegeneration, influencing both the onset and the course of dementia [47,48,49]. Mechanistically, vascular pathology disrupts cerebral blood flow, compromises the blood–brain barrier, and leads to ischemia, hypoperfusion, and microstructural white matter damage [7,9,10]. These changes do not act in isolation but interact with amyloid and tau pathology, accelerating cognitive decline and producing mixed dementia phenotypes that blur the boundaries between distinct syndromes [50]. The contribution of hypertension has been particularly well documented. Systematic reviews and meta-analyses by Forte and colleagues have demonstrated that elevated blood pressure, especially when sustained in midlife, significantly increases the risk of cognitive decline and dementia even in individuals without prior cerebrovascular events [7,9,10]. These studies highlight how hypertension impacts multiple cognitive domains, including executive functions, processing speed, and memory, and how the timely initiation of antihypertensive therapy can mitigate these risks. Untreated hypertension in late life has been shown to carry a much higher risk of dementia compared to treated hypertension, underscoring the critical importance of early and sustained intervention. The mechanisms linking hypertension to cognitive impairment include endothelial dysfunction, impaired neurovascular coupling, white matter hyperintensities, and reduced clearance of toxic proteins such as amyloid-β and tau [47,48,49,50]. These mechanisms demonstrate how vascular pathology not only causes vascular cognitive impairment but also synergistically accelerates neurodegenerative processes that are otherwise considered brain-first.

## 3. Comparative Pathophysiology of Brain-First and Body-First Trajectories

While the application of brain-first and body-first labels to individual neurodegenerative syndromes provides descriptive value, a central aim of this framework is to enable comparative mechanistic analysis across diseases. Viewed transversely, brain-first and body-first trajectories differ not only in the anatomical site of initiation, but also in molecular propagation dynamics, network vulnerability, and patterns of peripheral–central interaction [13,14,15,16,17,18,21,22].

In brain-first trajectories, the early accumulation of misfolded proteins within highly interconnected cortical and limbic hubs is expected to drive the rapid propagation of these proteins within the network, resulting in early cognitive vulnerability and relatively delayed peripheral involvement. This propagation is primarily constrained by synaptic connectivity and network topology, resulting in the early disruption of large-scale cognitive networks. In contrast, body-first trajectories are hypothesized to involve slower but more widespread propagation, which is constrained by autonomic and brainstem connectivity. Early peripheral and autonomic involvement may produce prolonged prodromal phases dominated by non-cognitive dysregulation, with central network destabilization emerging later as ascending pathways become engaged. These differences imply distinct causal chains linking molecular pathology, network architecture, and clinical expression, rather than merely different anatomical starting points. At the molecular level, brain-first trajectories are characterized by the early accumulation and propagation of misfolded proteins within intrinsically connected cortical and limbic networks, consistent with network-based models of neurodegeneration [20,21]. In these trajectories, protein spread appears to follow patterns of synaptic connectivity and selective neuronal vulnerability, with peripheral involvement emerging later as a downstream consequence of central pathology [21,22]. By contrast, body-first trajectories are hypothesized to originate in peripheral or autonomic compartments, where misfolded proteins or inflammatory signals may access the central nervous system via retrograde axonal transport along autonomic pathways, ultimately involving the brainstem and higher cortical regions [13,14,15,16,17,18].

Autonomic dysfunction represents a key mechanistic discriminator between trajectories. In body-first pathways, early alterations in autonomic regulation, circadian rhythms, and interoceptive signaling are prominent and may actively contribute to central network destabilization at prodromal stages [6,18]. Conversely, in brain-first trajectories, autonomic disturbances typically emerge later and are more plausibly interpreted as secondary consequences of degeneration within central regulatory hubs rather than primary drivers of disease initiation [16]. This comparative view suggests dynamic interactions. Peripheral inflammation, vascular dysfunction, and metabolic stress may amplify central vulnerability even in predominantly brain-first trajectories, while early central pathology may in turn accelerate peripheral dysregulation in body-first pathways [7,47,48,49,50]. This convergence provides a mechanistic explanation for mixed and overlapping phenotypes frequently observed across dementia syndromes. Framing brain-first and body-first models in this comparative manner generates testable analytical questions, including which combinations of central, peripheral, and autonomic biomarkers can prospectively distinguish trajectories, how early peripheral disturbances modulate network-level brain vulnerability, and whether trajectory-specific interaction patterns predict differential progression rates. Addressing these questions requires longitudinal, multimodal, and systems-oriented study designs. Taken together, these dimensions suggest that brain-first and body-first trajectories should be interpreted as dynamic configurations emerging from the interaction between initiation site, propagation constraints, and system-level coupling, rather than as fixed and mutually exclusive categories.

## 4. Biomarkers and Assessment Tools in Brain-First vs. Body-First Neurodegenerative Trajectories

Diagnosing and differentiating brain-first and body-first MCI phenotypes require comprehensive evaluations (See Table 1). If brain-first and body-first trajectories reflect distinct propagation dynamics, we would expect to see different temporal orderings of biomarkers rather than isolated markers. Brain-first trajectories would be expected to show early central protein abnormalities (e.g., AT(N) positivity, cortical hypometabolism, hippocampal atrophy) preceding detectable autonomic or peripheral alterations. Conversely, body-first trajectories would be expected to show early autonomic dysfunction, peripheral α-synuclein pathology, circadian disruption, or inflammatory signals before overt cortical involvement becomes measurable. In this view, trajectory classification depends on biomarker constellations and their temporal sequencing rather than on single diagnostic thresholds. Accordingly, prospective differentiation requires longitudinal assessment of both central and peripheral domains.

In brain first dementia, cerebrospinal fluid biomarkers provide direct insight into central pathological processes. Reduced Aβ42 indicates amyloid deposition, while elevated phosphorylated tau (p-tau) and total tau reflect neurofibrillary tangle burden and neuronal injury [51]. Plasma biomarkers, including p-tau181, p-tau217, and neurofilament light chain (NfL), are increasingly reliable surrogates for CSF, enabling minimally invasive monitoring of disease progression [35]. Emerging work also examines synaptic biomarkers (e.g., neurogranin) and inflammatory cytokines, which can provide early signals of synaptic dysfunction and neuroinflammation. MRI identifies early atrophy patterns in the hippocampus, entorhinal cortex, and temporoparietal regions [35,36,51]. Quantitative volumetrics and cortical thickness measurements allow longitudinal tracking of neurodegeneration. PET imaging, using amyloid tracers (PiB, Florbetapir) and tau tracers (flortaucipir/AV-1451), visualizes pathological protein deposition with regional specificity [20]. Functional imaging, such as resting-state fMRI and FDG-PET, assesses network-level alterations. Brain-first trajectories often show hypometabolism in hippocampal and default mode network regions, whereas subcortical networks remain relatively intact in the early stages [52]. EEG and MEG studies can detect early intra-cortical desynchronization and altered oscillatory activity, correlating with cognitive impairment severity. Neurodegeneration disrupts network connectivity. Functional connectivity analyses (fMRI, EEG, MEG) reveal reduced synchronization within and between memory, executive, and attentional networks [53,54]. These measures can serve as dynamic biomarkers of network integrity, capturing early cortical disconnection before overt atrophy or cognitive decline. Neuropsychological evaluation remains a cornerstone for detecting early central pathology in brain-first trajectories. These patients often present with isolated cognitive deficits, predominantly in episodic memory, reflecting early medial temporal lobe involvement [33,34,55]. When integrated with biomarker data, cognitive profiles allow differentiation from psychiatric conditions or vascular cognitive impairment, enhancing diagnostic specificity.

Body-first trajectories frequently manifest with early autonomic dysfunction, detectable via noninvasive tools. Heart rate variability (HRV) provides a quantitative index of autonomic tone; reduced HRV indicates impaired parasympathetic modulation and correlates with prodromal Parkinson’s disease and Lewy body dementia [56], as well as appears to be related to cognitive deficit in MCI [10]. Additional assessments include orthostatic blood pressure testing, sudomotor function assays, and gastrointestinal motility studies, which can identify early peripheral nervous system involvement before cognitive decline. Prodromal sleep disturbances are hallmark peripheral features in body-first trajectories. Polysomnography and actigraphy measure sleep architecture and circadian regularity. Circadian desynchronization between central and peripheral clocks may be an early indicator of multisystem pathology, potentially influencing neurodegenerative progression. Peripheral biopsies (skin, gut, salivary glands) can detect alpha-synuclein pathology in body-first phenotypes [40]. The gut microbiome is increasingly recognized as a modulator of neurodegeneration; dysbiosis may influence systemic inflammation, vagal signaling, and microglial activation, linking peripheral processes to central amyloid and tau pathology. Blood-based inflammatory markers and metabolites further provide insights into systemic contributions to neurodegeneration.

Within the present framework, biomarkers are not considered as isolated diagnostic markers, but as operational signatures that translate brain-first and body-first trajectories into empirically testable profiles. Distinct biomarker constellations are hypothesized to reflect different windows of vulnerability and mechanisms of progression, which in turn motivate trajectory-informed approaches to study design and intervention timing. From a comparative perspective, biomarker constellations can be interpreted as trajectory predictors rather than isolated diagnostic indicators. For example, early AT(N) positivity combined with preserved autonomic function may predict a brain-first trajectory characterized by rapid network-level propagation, whereas early autonomic dysfunction, reduced heart rate variability, and peripheral α-synuclein deposition may indicate a body-first trajectory with prolonged prodromal instability and delayed cortical involvement. To synthesize these findings, Table 2 maps the proposed brain-first and body-first signatures across biomarker, imaging, prodromal, and autonomic domains and also reports their current diagnostic performance.

### Body–Brain Coupling and Functional Synchronization: The New Era?

While the brain-first and body-first models primarily distinguish neurodegenerative trajectories based on the anatomical site of pathological initiation, they do not fully capture variations in systemic integration across central and peripheral processes. Within the present framework, body–brain coupling is conceptualized as a transversal third dimension that interacts with both brain-first and body-first trajectories, modulating their stability, propagation dynamics, and clinical expression. Rather than representing an alternative model, this dimension captures the degree of systemic integration across central, autonomic, and peripheral processes, thereby influencing how and when pathological changes become clinically manifest. For instance, early autonomic dysregulation may amplify central vulnerability even in predominantly brain-first Alzheimer’s disease, whereas impaired coupling in body-first trajectories may accelerate the transition from peripheral to cortical involvement, contributing to variability in prodromal duration and symptom expression. The concept of body–brain synchronization can be viewed as a mechanistic extension of this framework, addressing how failures in systemic integration may precede overt structural pathology. From this perspective, alterations in autonomic regulation, circadian rhythms, and interoceptive signaling do not represent an alternative model, but a complementary dimension that helps explain prodromal dysregulation across both brain-first and body-first trajectories. Early disruption of body–brain coupling may lower network resilience, modulate propagation dynamics, and shape the timing and expression of clinical symptoms.

The emerging evidence on body–brain synchronization therefore expands the traditional dual-path framework by suggesting that vulnerability may arise not solely from the site of pathological initiation but also from early disruptions in systemic coordination. This perspective enriches the conceptual value of the brain-first/body-first distinction and positions functional coupling as a potential third dimension capable of capturing early multisystem dysregulation that precedes structural neurodegeneration.

While the brain-first/body-first framework has provided an invaluable conceptual scaffold, it is essential to recognize its current limitations. First, much of the available evidence is associative or cross-sectional, making it difficult to establish the true temporal ordering of central versus peripheral pathology in human disease trajectories. Longitudinal, multimodal studies are scarce, and reverse causation cannot be excluded. Second, phenotypic heterogeneity within each disorder complicates the categorization of subtypes. Biomarker assays across CSF, plasma, and peripheral tissues lack full harmonization, and autonomic testing is often limited by variability in protocols and reference standards. These barriers make reproducibility and clinical translation challenging.

Moreover, it is plausible that the brain-first and body-first dichotomy oversimplifies reality. Rather than mutually exclusive categories, neurodegeneration may often reflect overlapping or convergent pathways, with central and peripheral insults interacting dynamically across disease stages. For instance, systemic inflammation or vascular dysfunction may accelerate amyloid or tau pathology, even in primarily brain-first Alzheimer’s disease, while peripheral α-synucleinopathy may coexist with early cortical changes in Parkinson’s disease or dementia with Lewy bodies.

A key motivation for this review stems precisely from these limitations. Despite the proliferation of studies investigating central and peripheral contributions to neurodegeneration, the literature remains fragmented across disorders, methods, and biomarkers. By bringing together evidence from neuropathology, neuroimaging, autonomic physiology, and peripheral tissue analysis, this review offers a unified synthesis intended to contextualize these findings within a single trajectory-based framework. Such integration is necessary to overcome the conceptual silos that have historically hindered cross-disease comparisons and early diagnostic stratification.

Conceptually, we propose that disrupted body–brain synchronization represents a complementary dimension to the brain-first/body-first framework. This perspective emphasizes early failures in systemic coordination—across autonomic, circadian, and network-level domains—as a potential driver of vulnerability to neurodegeneration.

**Figure 1:**
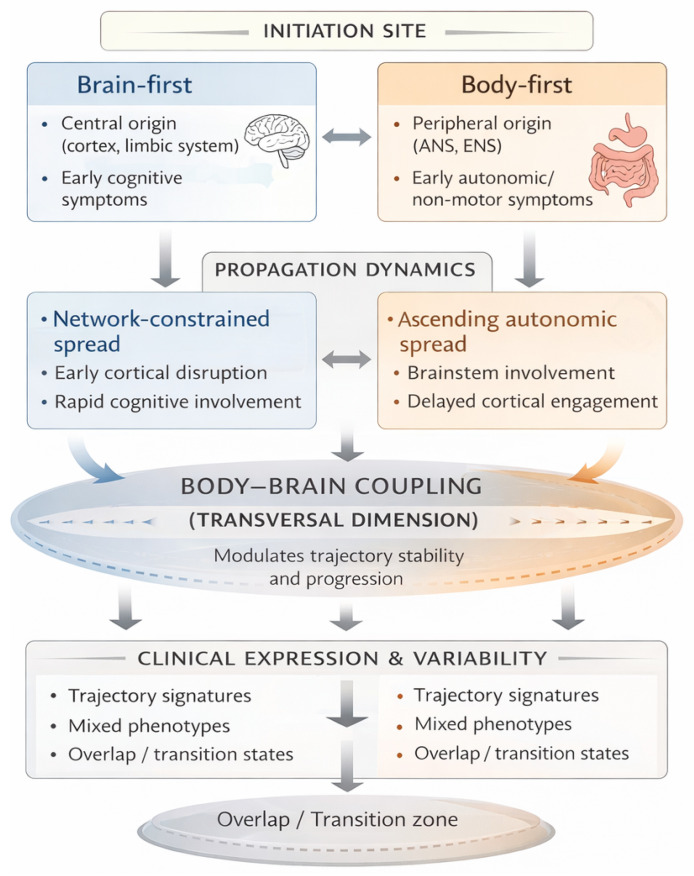
Conceptual framework.

Conceptual framework (Figure 1) integrating brain-first and body-first neurodegenerative trajectories across three levels: initiation site, propagation dynamics, and body–brain coupling. Body–brain coupling is represented as a transversal dimension modulating trajectory stability and progression. The model emphasizes that trajectories emerge as dynamic configurations, with overlap and transition zones rather than fixed categories.

## 5. Therapeutic Implications

A key translational implication of the trajectory-based framework concerns the interpretation of biomarker constellations in relation to therapeutic timing and biological targets. If brain-first and body-first trajectories differ in their sites of initiation and propagation dynamics, then biomarker signatures should not only serve diagnostic purposes but may also signal different windows of vulnerability for intervention.

For example, early AT(N) biomarker positivity combined with hippocampal network disruption would be consistent with a brain-first trajectory and may indicate a stage in which interventions targeting central protein aggregation, synaptic resilience, or network plasticity are biologically plausible. Conversely, constellations characterized by autonomic dysfunction, reduced heart rate variability, peripheral α-synuclein deposition, or circadian dysregulation may reflect body-first trajectories in which peripheral or systemic processes contribute substantially to disease initiation and early propagation.

Within this perspective, biomarkers function not only as diagnostic indicators but as operational signals of underlying biological dynamics, helping to identify phases in which central versus systemic processes may be more relevant targets for experimental intervention strategies.

Within this framework, therapeutic implications are considered at the level of biological logic rather than clinical prescription. While the brain-first/body-first distinction does not specify treatments, it does constrain the range of plausible intervention targets, timing, and mechanisms across disease stages.

The brain-first and body-first distinction is not intended to prescribe particular interventions, but to provide a biological rationale for stratification, timing, and mechanism-oriented interpretation of therapeutic strategies across neurodegenerative syndromes. In brain-first trajectories, exemplified by Alzheimer’s disease and frontotemporal dementia, early central nervous system involvement suggests that vulnerability arises primarily within large-scale cortical and limbic networks. Conceptually, this implies that research strategies may benefit from prioritizing the early identification of central network disruption and aligning interventions—pharmacological or non-pharmacological—with stages in which central plasticity and compensatory capacity are still preserved [40,57,58,59]. This perspective emphasizes biological context and timing. Conversely, body-first trajectories, including dementia with Lewy bodies and Parkinsonian dementias, are characterized by prominent early peripheral and autonomic involvement. Within this framework, peripheral dysfunctions such as autonomic imbalance, sleep disturbances, or gut–brain axis alterations are not viewed merely as secondary features, but as integral components of the disease trajectory [13,14,15,60,61,62]. Conceptually, this suggests that trajectory-informed approaches may place greater emphasis on early peripheral and systemic targets, multimodal assessment, and interventions addressing body–brain interactions alongside central pathology [63]. Crucially, the brain-first/body-first framework does not imply mutually exclusive therapeutic pathways. It suggests how differences in sites of initiation and propagation dynamics may shape windows of vulnerability and responsiveness across disease stages. In this sense, the framework provides a lens through which heterogeneous responses observed across studies and patient populations can be interpreted, without requiring post hoc attribution to specific treatments or trial outcomes. Beyond pharmacological considerations, the framework also underscores the potential relevance of non-pharmacological strategies tailored to disease trajectories. Interventions targeting cognitive networks, lifestyle factors, autonomic regulation, and systemic physiology may differentially interact with brain-first and body-first pathways [64,65]. While empirical validation is required, such approaches illustrate how trajectory-based stratification can inform comprehensive and individualized research and care models without reducing therapeutic implications to drug-centric solutions. In this sense, the framework links biomarkers to intervention logic without overstepping evidentiary boundaries. Differences in initiation sites and propagation dynamics are expected to influence windows of vulnerability, compensatory capacity, and responsiveness to system-level modulation. Articulating these links explicitly is a necessary intermediate step between descriptive pathology and clinical translation. To illustrate the operational logic of this framework: a profile characterized by early AT(N)+ positivity, hippocampal atrophy, and preserved autonomic function may be conceptually consistent with a brain-first trajectory, in which central mechanisms and network-level vulnerability are likely to dominate early disease dynamics; conversely, a constellation marked by autonomic dysfunction, reduced heart rate variability, REM sleep behavior disorder, and peripheral α-synuclein deposition may reflect a body-first trajectory, where peripheral and systemic processes are more plausibly implicated in the initial phases of disease propagation.

## 6. Limitations of the Study

Several limitations of the present review should be acknowledged.

First, much of the current evidence supporting brain-first and body-first trajectories remains associative rather than causal. Many studies rely on cross-sectional observations or retrospective reconstructions of prodromal symptoms, making it difficult to establish definitive temporal ordering between peripheral and central pathology.

Second, biomarker evidence remains heterogeneous and not fully harmonized across studies. Differences in assay methodologies, thresholds, and sampling protocols complicate direct comparison across cohorts and limit the reproducibility of some peripheral biomarkers. Third, while the present work proposes a conceptual integration of central, peripheral, and autonomic evidence, the review is not designed as a clinically prescriptive framework. The framework is intentionally positioned at a mechanistic and hypothesis-generating level rather than proposing trial-ready stratification algorithms. Fourth, the literature itself remains fragmented across diseases, biomarkers, and methodological traditions. As a result, some elements of the proposed framework should be interpreted as conceptual integration rather than established empirical consensus. For these reasons, the brain-first/body-first model should be regarded as a heuristic and generative framework, intended to guide future longitudinal and multimodal research rather than as a definitive classification scheme.

## 7. Conclusions

The brain-first versus body-first framework provides a unifying conceptual lens for interpreting the marked heterogeneity of dementia syndromes. It emphasizes that neurodegeneration is not a uniform process, but rather unfolds along partially distinct biological trajectories. Mixed or ambiguous trajectories should not be considered classification failures, but rather as the expected outcomes of the interaction between central and peripheral processes that unfold over time. Trajectory instability and drift may reflect dynamic re-weighting of central versus systemic drivers, rather than discrete subtypes. By integrating neuropathological evidence, clinical prodromes, and biomarker signatures, this perspective moves beyond disease labels toward a trajectory-based understanding of cognitive decline that explicitly incorporates both central and peripheral drivers. At the same time, it is essential to acknowledge the limitations of a binary framework when applied to a complex and likely continuous disease spectrum. Brain-first and body-first trajectories should not be interpreted as mutually exclusive categories, but as heuristic anchors along a multidimensional continuum. Mixed and overlapping pathways are likely common, particularly in advanced disease stages or in conditions with combined pathologies, and current evidence does not yet support reliable antemortem classification at the individual level. The absence of validated diagnostic criteria, heterogeneity of biomarker assays, and limited longitudinal data constrain immediate clinical translation and underscore the provisional nature of the model. These limitations define the current boundaries of the framework that could be overcome. Viewed in this way, the brain-first and body-first distinction yields several testable hypotheses and methodological priorities for future research. First, longitudinal cohort studies should prioritize the concurrent acquisition of central and peripheral measures from the prodromal stage onward, integrating central protein biomarkers, autonomic testing, peripheral tissue markers, and physiological indices of body–brain coupling. Such designs are essential to establish temporal ordering and to determine whether peripheral dysfunction actively modulates central network vulnerability. Second, future studies should explicitly test whether disrupted body–brain synchronization—manifested through alterations in autonomic regulation, circadian rhythms, and interoceptive signaling—predicts differential progression patterns across trajectories. This hypothesis reframes neurodegeneration as a disorder of systemic network integration rather than isolated regional pathology. Third, trajectory-based models predict that phenotypic heterogeneity arises from dynamic interactions between central and peripheral processes. Methodologically, this requires data-driven stratification approaches capable of capturing hybrid or evolving trajectories, moving beyond rigid diagnostic boundaries. Finally, advancing this framework will require harmonized, longitudinal, and systems-oriented research infrastructures capable of capturing real-world heterogeneity. Only through such approaches can the brain-first and body-first models evolve from conceptual scaffolds into empirically grounded tools that refine our understanding of dementia biology and guide future precision-oriented research. In summary, the brain-first and body-first framework should be regarded as a flexible integrative model rather than a definitive classification scheme.

## Figures and Tables

**Table 1:** Example and classification of brain-first/body-first phenotype.

Domain	Brain-First Phenotype	Body-First Phenotype
**Temporal** **sequence**	Central pathology precedes peripheral involvement	Peripheral/autonomic dysfunction precedes central involvement
**Prodromes**	Cognitive impairment, subtle motor/behavioral changes	Hyposmia, constipation, orthostatic hypotension
**Primary** **pathology**	Hippocampus, cortex, basal ganglia	ENS, ANS (gut, olfactory epithelium, cardiac plexus)
**Proteinopathy**	Tau, amyloid-β, alpha-synuclein (central accumulation)	Alpha-synuclein in peripheral tissues, retrograde spread to CNS
**Biomarker profile**	Early AT(N) positivity, hippocampal atrophy, cortical hypometabolism, nigrostriatal loss	HRV reduction, abnormal cardiac MIBG, peripheral alpha-synuclein biopsies, and later cortical changes
**Imaging**	Early cortical/hippocampal changes	Later cortical/hippocampal changes, earlier brainstem/autonomic involvement
**Examples**	Alzheimer’s disease, frontotemporal dementia	Parkinson’s disease (body-first subtype), dementia with Lewy bodies (body-first subtype), vascular dementia

**Table 2:** Comparative brain-first and body-first signatures across biomarker, imaging, prodromal, and autonomic domains, including qualitative diagnostic performance and major limitations.

Domain	Brain-First Signature	Body-First Signature	Evidence Strength & Validation	Supporting Evidence and Major Gaps
**Core** **biomarker**	CSF Aβ42 ↓, p-tau ↑, t-tau ↑; plasma p-tau181/p-tau217, NfL; synaptic markers (neurogranin); inflammatory cytokines	Peripheral α-synuclein in skin/gut/salivary gland biopsies; blood-based inflammatory markers; microbiome/metabolite profiles	Brain-first: +++ (clinical validated)|Body-first: +/++ (research-supported/emerging)	CSF/AT(N) highly validated; plasma p-tau promising but thresholds & comparability remain issues; peripheral α-syn biopsies variable sensitivity/specificity
**Neuroimaging**	MRI: hippocampal, entorhinal, temporoparietal atrophy; PET amyloid/tau positive early; FDG-PET: hippocampal/DMN hypometabolism; fMRI/EEG/MEG: early cortical desynchronization	Early relative sparing of cortex; later cortical changes; autonomic/circadian alterations	Brain-first: +++ (clinical validated)|Body-first: ++ (research-supported)	MRI + PET: strong diagnostic value; EEG/MEG supportive; peripheral/circadian measures emerging with lower validation
**Prodromal features**	Cognitive-first: episodic memory deficits → progressive executive/attentional decline	Early non-cognitive: sleep behavior disorder, hyposmia, constipation, orthostatic hypotension, reduced HRV, circadian dysregulation	Brain-first: +++ (clinical validated)|Body-first: ++/+++ (research-supported/partially clinical)	Neuropsychology well-established; RBD polysomnography high predictive value for α-synucleinopathies
**Autonomic tests**	Typically preserved early; later orthostatic intolerance or HRV changes	Early abnormalities: HRV reduction, orthostatic hypotension, sudomotor/gastrointestinal dysfunction	Brain-first: + (emerging)|Body-first: ++ (research-supported)	HRV and BP testing: moderate specificity, valuable in prodromal PD/DLB
**Pathology trajectory**	Initiation in hippocampus/DMN → cortical spread → later subcortical/peripheral involvement	Initiation in ANS/ENS/olfactory → brainstem → cortical spread	Brain-first: +++ (neuropathological validated)|Body-first: ++ (research-supported)	Brain-first staging robust (Braak, AT(N)); body-first trajectory supported in synucleinopathies
**Overall**	Central initiation with cognitive-first prodrome	Peripheral/autonomic initiation with non-cognitive prodrome	Brain-first: +++ (well-established)|Body-first: ++ (emerging/partial validation)	Brain-first supported by robust biomarker/imaging frameworks; body-first supported in PD/DLB, emerging in mixed phenotypes

CSF = cerebrospinal fluid; Aβ42 = amyloid-beta 42; p-tau = phosphorylated tau; t-tau = total tau; NfL = neurofilament light chain; PET = positron emission tomography; FDG-PET = fluorodeoxyglucose PET; DMN = default mode network; fMRI = functional MRI; EEG = electroencephalography; MEG = magnetoencephalography; HRV = heart rate variability; RBD = REM sleep behavior disorder; BP = blood pressure; ANS = autonomic nervous system; ENS = enteric nervous system; PD = Parkinson’s disease; DLB = dementia with Lewy bodies; AT(N) = amyloid/tau/neurodegeneration framework; MRI = magnetic resonance imaging. Strength of evidence is reported using a semi-quantitative scale (+ = preliminary; ++ = moderate; +++ = strong). ↓: lower, ↑:higher. Level of validation refers to the degree of clinical and methodological consolidation of each biomarker domain.

## Data Availability

No new data were created or analyzed in this study. Data sharing is not applicable to this article.

## Appendix A

This article was conducted as a narrative review in accordance with the SANRA (Scale for the Assessment of Narrative Review Articles) guidelines [65]. Studies were identified through searches in PubMed, Web of Science, and Scopus, with additional seminal references included regardless of publication date. Search terms combined disease entities (e.g., Alzheimer’s disease, Parkinson’s disease, dementia with Lewy bodies, frontotemporal dementia) with model descriptors (e.g., brain-first, body-first, autonomic dysfunction, prodrome, biomarkers). The review is selective and hypothesis-driven rather than systematic. Priority was given to conceptual frameworks, neuropathological staging studies, multimodal neuroimaging investigations, autonomic and peripheral biomarker research, and longitudinal prodromal cohorts that inform trajectory-based models of neurodegeneration.
